# Reply to the letter of Braber et al.: ‘Coronary stenting versus bypass surgery in elderly with multivessel disease: long-term mortality rate is still up for debate’

**DOI:** 10.1007/s12471-020-01520-z

**Published:** 2020-11-13

**Authors:** M. E. Gimbel, L. M. Willemsen, J. M. ten Berg

**Affiliations:** Department of Cardiology and Cardiothoracic Surgery, St. Antonius Hospital, Nieuwegein, The Netherlands

We would like to thank Braber et al. [1] for this opportunity to discuss the results of our study regarding the follow-up of elderly patients with multivessel disease after revascularisation. As stated in the discussion section of our article, we were aware that the design of the study is inherent to risk of bias when comparing two strategies. However, we chose an observational study design on purpose to describe the long-term follow-up in an unselected population. Even though a randomised controlled trial (RCT) may be the best method to approach the most genuine answer regarding the best revascularisation strategy in elderly with multivessel disease, this would require equipoise from the Heart Team’s perspective between coronary artery bypass grafting (CABG) and percutaneous coronary intervention (PCI), which is rarely the case, especially in this elderly population. Also, physicians tend to know what is best for their patients and therefore do not advise them to be candidates for an RCT. Furthermore, of all patients screened, only a minority is included in an RCT; thus, a highly selected population is studied, which does not reflect the patients we care for every day.

This was for instance shown in the SYNTAX RCT (CABG vs PCI in multivessel disease), in which only a small percentage of the patients presented to the Heart Team was eventually randomised in the study [2]. In addition, we argue that patients with variables such as cancer, dementia or frailty are unlikely to be randomised in such a trial for reasons listed above. The fact that, as of today, an RCT has not been performed in the elderly, demonstrates the complexity of carrying out this trial in elderly frail patients, who often have many comorbidities. Still, we agree with Braber et al. that the comparison of the two strategies includes confounding, even after our efforts to correct for this. We could have placed more emphasis on this limitation in the conclusion of our article.

Braber et al. also indicated that our method of correction for confounders at baseline was confusing. Therefore, we would like to explain that we included all baseline variables with a *p*-value <0.1 in the univariate analysis for the multivariate analysis per outcome, as is explained in the method section of our article. Variables in the multivariable model that did not affect the outcome with *p* > 0.05 were successively excluded from the model, which resulted in a final model of significant variables influencing the outcome and correcting for them. We created a different model per outcome and subsequently corrected for the significantly different variables from baseline. For example, if age, diabetes and previous CABG had a *p*-value <0.1 in the univariate analysis, these variables were included in all multivariate analyses. If age was not a significant variable in the multivariate analysis for myocardial infarction, it was excluded from the model for that outcome, resulting in a correction for diabetes and previous CABG for the outcome ‘myocardial infarction’. We agree with Braber et al. that variables such as cancer, neurologic disorders (e.g. dementia) and frailty are interesting and important to correct for. However, in order to do so, a prospective registry should be performed.

The trials mentioned by Braber and colleagues (BARI, FREEDOM and EXCEL) comparing PCI with CABG have their own limitations with regard to the elderly population at hand. The BARI trial, performed in 1995, investigated percutaneous transluminal balloon angioplasty and is therefore not applicable to this discussion [3]. In addition, because the mean age in these RCTs was approximately 65 years, these characteristics (age and percutaneous transluminal balloon angioplasty) complicate translating data from these RCTs to our study population of elderly with multivessel disease, which emphasises the need for contemporary data on these patients.

Lastly, we share the opinion that PCI is progressively improving and becoming more effective in the treatment of coronary artery disease. In addition, antiplatelet treatment continues to improve and is being tailored to specific patient characteristics, thereby reducing complications of PCI, such as stent thrombosis but also bleeding in the elderly population. That was one of the reasons for our contemporary analysis. Despite improved antithrombotic therapy for PCI patients, the results of our observational study did not substantiate a more favourable outcome after PCI compared with CABG. We do, however, agree with Braber et al. that this difference may not be due to the failure of PCI, but due to the PCI patients in our study being frailer and having more comorbidities than the CABG patients.
